# All eyes on you: how researcher presence changes the way you walk

**DOI:** 10.1038/s41598-020-73734-5

**Published:** 2020-10-13

**Authors:** Kenzie B. Friesen, Zhaotong Zhang, Patrick G. Monaghan, Gretchen D. Oliver, Jaimie A. Roper

**Affiliations:** School of Kinesiology, Auburn University, Auburn, USA

**Keywords:** Health care, Health occupations, Medical research

## Abstract

Most human movement research takes place within controlled laboratories where researchers observe participant movement. Because a majority of daily activity is performed without observation, we hypothesized movement within a laboratory would vary when there was a small, large or absence of research group. We also hypothesized that personality type would influence movement during observation. Participants completed a personality questionnaire, then walked in a laboratory during three different conditions: no research group (no researchers), small research group (2 researchers), and large research group (6–10 researchers). Results revealed spatiotemporal parameters were altered between conditions, however personality type did not influence changes in movement. As the number of researchers increased, gait speed, cadence, and stride length increased, and step duration decreased. Gait speed increased by .03 m/s from the no research group to the small research group, by .06 m/s from the no research group to the large research group, and by .03 m/s from the small to large research group (all *p* values < .001). Understanding how researcher observation modifies movement is important and affects the replicability of results, as well as the interpretation of laboratory-based movement studies to activities of daily living in real world settings.

## Introduction

Walking is the most common form of human locomotion, and as such, remains a popular outcome in assessing movement pathologies^1^. Gait research is regularly completed in a laboratory setting, yet most of a person’s daily walking occurs outside of the laboratory, during activities of daily living. Currently, much of the in-laboratory findings are interpreted and translated to daily walking in a real-world setting, although this comparison assumes that gait in the laboratory setting is consistent with normal day-to-day gait^2–4^.

Assuming gait performance in an observational setting, such as a doctor’s office, rehabilitation clinic, or research laboratory mimics real-world walking may not be accurate. This oversight can result in misdiagnosis of comorbidities and disease, as well as therapeutic prescriptions, which regularly depend on gait speed and other gait parameters for at-risk identification^5,6^. One noteworthy difference between the real-world and an observational setting is the presence of experts who scrutinize movement. Research-based studies commonly require a participant to perform movements while being watched by laboratory members; therefore, much of the research taking place in the laboratory setting is under intent observation. Often in educational and training environments, laboratories are more populated with additional personnel, consisting of both instructors and student learners. Studies examining older and at-risk populations often require additional laboratory support as a safety precaution, leading to additional observers being present during data collection. Similarly, studies examining high level performing athletes often garner a larger set of observers due to piqued interest. Also, in academic settings, biomechanics laboratory space is regularly designed to allow for workstations along the perimeter, increasing the number of individuals present during data collection.

Although research is regularly completed in the presence of many researchers and observers, research has shown that the presence of observers can lead to alterations in behavior and can influence task performance, commonly referred to as the Hawthorne effect^7–9^. Research has also shown that the feeling of being judged, evaluated, or having one’s actions approved or disapproved by observers may also lead to alterations in movement^10^. This environment of judgment and scrutiny is often unintentionally created when gait is assessed in front of a group of researchers. However, the degree of discomfort that may result from a large group of researchers, and subsequent gait alterations is influenced by an individual’s personality type^11^. Personality has previously been shown to influence parameters of gait^11^, and recent work has indicated personality traits examined through the Big Five personality test relates to movement in gait^12^. However, no previous study has assessed whether the combination of personality and the effects of researchers interact to alter gait. For this reason, the current study has included measures of personality while assessing the effects of researcher presence on gait spatiotemporal parameters.

Prior studies have acknowledged the effects of observation on gait in older, unhealthy^4,9,13^, and injured populations^2,14^. Similarly, awareness of data collection affected gait kinematics during individual versus continuous trials, suggesting study conditions also affect the measured outcomes during analysis^15^. While the nature of the research protocol affects gait outcomes, no study has examined the influence of observers on walking performance. It is critical to understand how small and large sizes of research groups may impact movement performance, and subsequently influence the translation of laboratory findings to the real-world.

Research has demonstrated a weak correlation (r = 0.333) between in-laboratory and real-world gait speed^4^. Differences in movements produced in an observational setting may be limiting the translation of research results to real-life settings outside the laboratory. Potential modifications in laboratory movement may also be influenced by personality type, as research shows personality affects parameters of gait^11^. Therefore, our primary goal was to understand how research group size affects spatiotemporal parameters of gait. We hypothesized that larger sized research groups with an increased number of observers, atypical of daily gait, would influence spatiotemporal parameters. A subsequent purpose was to examine potential alterations in spatiotemporal parameters of gait between the first and last few strides of the trial. Finally, our third aim was to examine if personality measures were related to changes in gait according to the size of the research group. We expected those with higher extroversion traits would exhibit less alterations in gait. While research has shown variance in gait between laboratory and real-world settings, the presence of an intent group of researchers may be the reason for the conflicting results.

## Results

No significant correlations were observed between the participant’s personality scores and gait speed. Further, no significant correlations were found between personality measures and the change in spatiotemporal parameters based on research group size; therefore, personality measures were not used further in the analyses.

**Figure 1:**
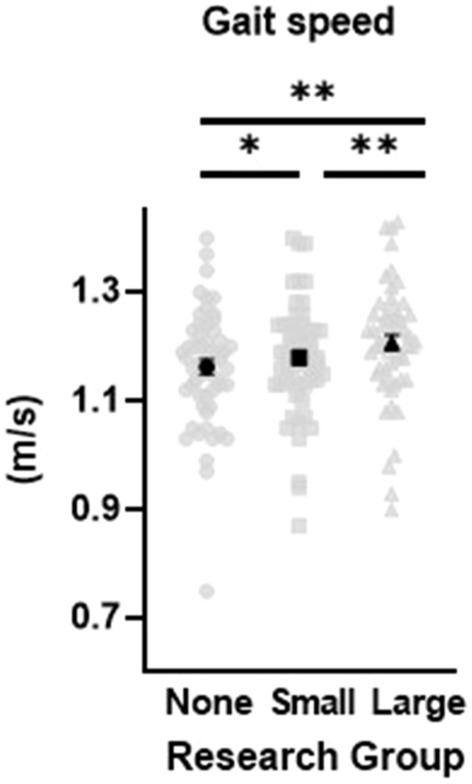
Gait speed is increased in the presence of increased number of researchers. Light data points reflect individual values while the dark data points represent mean values. Error bars represent standard error measurement. *denotes statistical significance with *p* ≤ .01 and **denotes statistical significance with *p* < .001.

**Figure 2:**
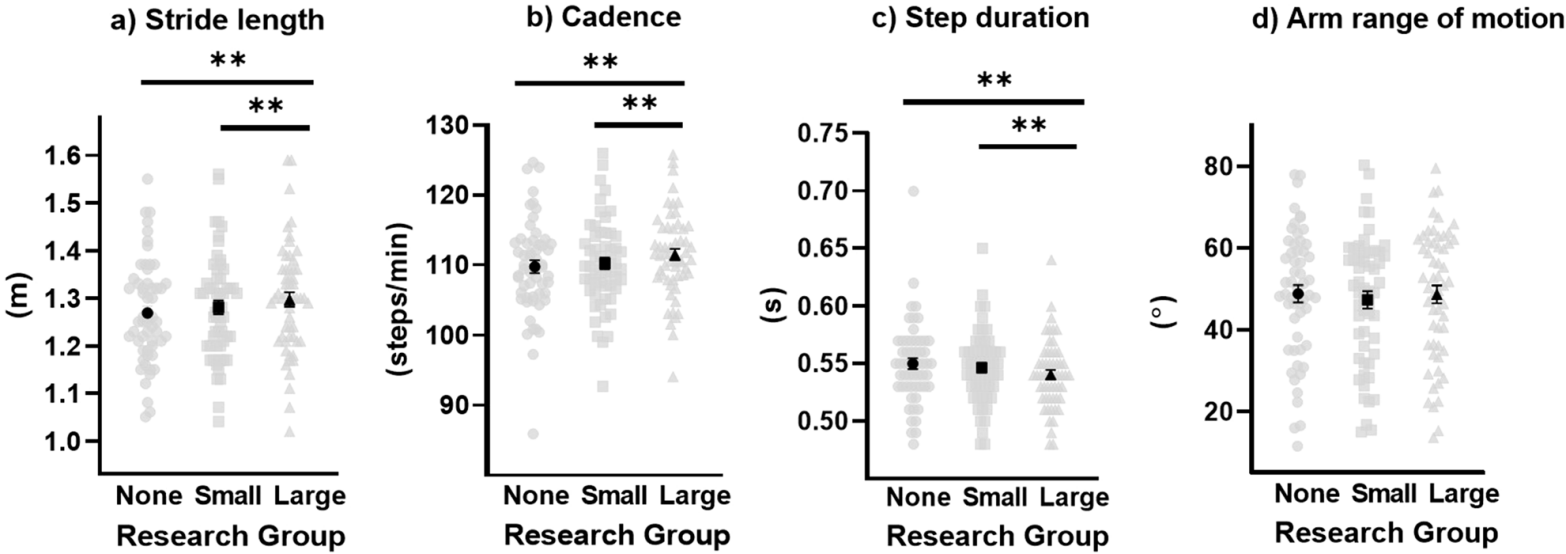
Stride length, cadence, and step duration all have significant differences between the no research group and the large research group, as well as significant differences between the small research group and the large research group. Arm range of motion shows no statistically significant differences. Light data points reflect individual values while the dark data points represent mean values. Error bars represent standard error measurement. **denotes statistical significance with *p* < .001.

Means and standard deviations for all spatiotemporal parameters for each research group are reported in Table 1. The one-way multivariate analysis of variance (MANOVA) was statistically significant between condition (research groups), F(10,46) = 10.16, *p* < 0.001, η^2^_*p*_ = 0.688, with an alpha level set at 0.05. Univariate analysis revealed gait speed (*p* < 0.001, η^2^_*p*_ = 0.421), cadence (*p* < 0.001, η^2^_*p*_ = 0.364), stride length (*p* < 0.001, η^2^_*p*_ = 0.336), and step duration (*p* < 0.001, η^2^_*p*_ = 0.320), were different between research groups, while arm range of motion (*p* = 0.070, η^2^_*p*_ = 0.047) was not (Figs. 1, 2). As the number of researchers increased, gait speed, cadence, and stride length increased, step duration decreased, and arm range of motion did not change. Gait speed, cadence, step duration, and stride length revealed differences between the no research group and the large research group, as well as between the small research group and the large research group. Gait speed was the only variable to also show differences between the no research group and the small research group.

**Table 1:** Means and standard deviations for all spatiotemporal variables per research group condition (mean ± standard deviation).

	No research group (no researchers)	Small research group (2 researchers)	Large research group (6–10 researchers)
Gait speed (m/s)	1.16 ± .11	1.19 ± .09	1.22 ± .10
Cadence (steps/min)	110 ± 7	110 ± 6	111 ± 6
Step duration (s)	.55 ± .04	.55 ± .03	.54 ± .03
Stride length (m)	1.27 ± .11	1.28 ± .11	1.30 ± .11
Arm ROM (º)	48.9 ± 15.7	47.4 ± 15.8	48.7 ± 16.1

**Figure 3:**
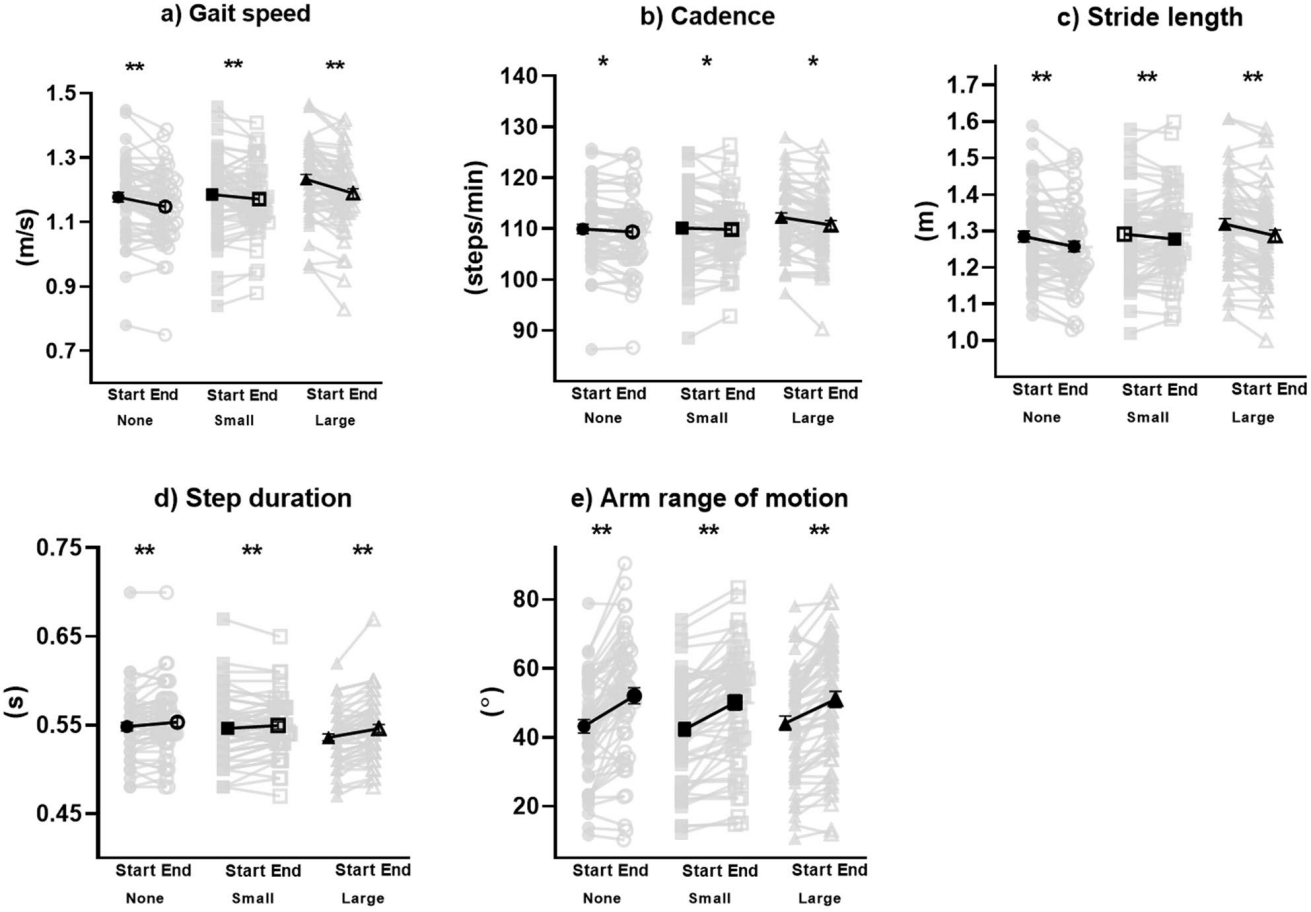
All spatiotemporal parameters revealed statistically significant differences between the start and end of each trial. Light data points reflect individual values while the dark data points represent mean values. Error bars represent standard error measurement. *denotes statistical significance with *p* < .01. **denotes statistical significance with *p* < .001.

Comparing the first few strides to the last few strides within trial, there was not a statistically significant interaction with research group (*p* = 0.059). Main effect for portion of trial, F(5,51) = 41.15, *p* < 0.001, η^2^_*p*_ = 0.801, and for research group, F(10,46) = 11.81, *p* < 0.001, η^2^_*p*_ = 0.720, were both significant. Main effect of research group revealed gait speed (*p* < 0.001, η^2^_*p*_ = 0.459), cadence (*p* < 0.001, η^2^_*p*_ = 0.383), stride length (*p* < 0.001, η^2^_*p*_ = 0.384), and step duration (*p* < 0.001, η^2^_*p*_ = 0.365) were different (over the entire trial) between research group, but arm range of motion (*p* < 0.081, η^2^_*p*_ = 0.085) was not. Univariate follow up analysis revealed all five variables, gait speed (*p* < 0.001, η^2^_*p*_ = 0.337), cadence (*p* = 0.004, η^2^_*p*_ = 0.145), stride length (*p* < 0.001, η^2^_*p*_ = 0.387), step duration (*p* < 0.001, η^2^_*p*_ = 0.305), and arm range of motion (*p* < 0.001, η^2^_*p*_ = 0.616) were different between the first and last few strides within trial (Fig. 3). Specifically, compared to the first few strides, gait speed (mean difference = 0.03 m/s), cadence (mean difference = 1 step/min) and stride length (mean difference = 0.02 m) decreased, while step duration (mean difference = 0.01 s) and arm range of motion (mean difference = 7.9º) increased during the last few strides across all groups.

## Discussion

This study aimed to examine the influence of researcher observation on spatiotemporal parameters during gait. Our main findings reveal that: (1) the number of researchers significantly influenced spatiotemporal parameters of gait, importantly gait speed, (2) the large research group had the greatest impact on spatiotemporal parameters compared to the no research group, (3) spatiotemporal parameters changed significantly from the first to the last few strides within each research group and (4) personality measures were not related to gait performance during different research group conditions.

Our results show that gait speed, cadence, and stride length increase while step duration decreases in the presence of a large group of researchers. These findings suggest that explicit observation by others prompted altered spatiotemporal parameters during data collection. The alterations experienced between research group trials could be a contributing mechanism leading to modified movement in the laboratory compared with real-world movement, as research has shown in-laboratory gait speed did not correlate well with daily gait speed outside the laboratory^4^. Most notably, gait speed is commonly used as a clinical assessment tool and recent research has even referred to it as the “sixth vital sign,” alluding to the high credibility the measure has in determining functional ability^5,6,16,17^. Gait speed is inherently easy to measure, especially through the use of simple and clinical movement analysis tools such as wireless inertial sensors. With self-selected gait speed becoming an increasingly popular assessment tool^5^, gait speed must be accurately measured. In some clinical and educational settings, it is common for numerous researchers and students to be present at such assessments for learning purposes. Our results suggest added researchers in a laboratory may be affecting the replicability of spatiotemporal measures during gait in and out of laboratory settings.

Previous work has shown a 0.08 m/s difference in gait speed is considered clinically significant^18,19^. Our data revealed gait speed increased by 0.03 m/s from the no research group to the small research group, by 0.06 m/s from the no research group to the large research group, and by 0.03 m/s from the small research group to the large research group. Therefore, it is important to note that although our statistical results were significantly different, they were not of clinical significance. Particular attention should be drawn to the slight variances in step duration. Although differences were statistically significant, there was only a minuscule change in mean step duration between the small and large research groups. As a result, caution should be taken when interpreting results, especially due to the homogeneity of our population.

While a previous study examining an older population demonstrated clinically meaningful differences in gait while in a laboratory environment opposed to a real-world setting^4^, the current study population comprised of healthy young adults revealed no clinically significant changes. Quantifying changes in gait due to the presence of researchers is especially important in patient populations who are aging, unhealthy, and have a greater need of clinical assessment. Because increased gait speed is associated with healthier participants^1^, a clinician risks falsely reporting a patient as healthy if there is a large group of observers present. However, understanding these alterations in a normal, healthy population helps isolate the effects of researcher presence while limiting outside factors such as age and comorbidities. Given the lack of clinical differences and the small variability between conditions, we are unable to comment further on whether a less homogenous population would result in clinically significant results.

A distinct difference between normal daily walking and in-laboratory walking is the controlled atmosphere of the laboratory. In such a setting, there are usually researchers present examining and observing the movement. This observation may alter movement, as theories such as the Hawthorne effect acknowledge a person’s changed behavior or movement in response to visual observation^9^. While most human movement research takes place in a laboratory setting with researchers present, results may be altered due to this aforementioned effect. Performance changes induced by the Hawthorne effect may be either good or bad. For example, it may be important to mimic a game-like audience when measuring athletic and sport performance. In this case, a large audience may be more appropriate than a small audience. Conversely in research which seeks to replicate daily activity in a real-world environment, a smaller group of researchers may be more suitable. Considering the participants in the current study were informed that the researchers present during the collection were “experts,” and would be closely analyzing their gait as they walked, our results may also be explained by Cottrell’s evaluation apprehension theory^10^. Participant performance may have been affected not only because of the presence of others, but also because our group of researchers contained specific knowledge required to assess and scrutinize the movement^10^. We must acknowledge that the differences observed in our study were small, suggesting that studies investigating healthy young individuals may be less at risk for large changes in overall results.

Our results suggest that a significant portion of altered movement occurs when a large number of researchers are present compared to no researchers. Previous work has shown that actors report higher anxiety in front of larger crowds, implying that audience size affects movement and emotional patterns^20^. Similarly, musicians report changes in timing and coordination^20,21^ when in front of an evaluative audience. Controlling for research group size during the analysis of “normal” movement may decrease unwanted error and improve reproducibility and interpretation of results into a normal setting where intent observation is less prominent and frequent. Perhaps in-laboratory human movement studies should further consider reporting or limiting the number of researchers present during a data collection to control for the variability that may arise from the pressure of observation. Controlling for the number of researchers is especially important in a mixed or repeated trial analysis when comparing within participants and between days and trials. Maintaining research group size may also help to control for variability between trial, participants, and across studies. A potential limitation of the current study was the variability in size of the large research group. Our large research group ranged from 6–10 individuals, with the majority being 8–10 researchers per research participant. Our number of researchers changed based on availability and scheduling of laboratory members. The current study found the large research group prompted the most significant changes in parameters, suggesting limiting data collection to a smaller group of researchers may improve the consistency of walking styles in and out of the laboratory.

Comparing the first and last few strides of each research group, gait speed, cadence, and stride length decreased towards the end of the trial, while step duration and arm range of motion increased (Fig. 3). Our results support the idea that greater acclimatization may diminish the effects of researcher presence; however, whether the influence of researcher presence has already been diminished by the end of the current study’s trials is still unknown. Comparison of the first few strides and the last few strides between conditions is needed to investigate the amount of familiarization necessary to minimize the influence of researcher presence. Previous work has reported that during treadmill walking, knee kinematics and most spatiotemporal parameters reached steady state after 30 s while stride length required 10 min^22^. Similar findings may exist for over ground walking adaptation under the influence of research observation; different parameters may require varied time for acclimatization before reaching steady state. Future studies should determine if different acclimatization strategies exist and may need to change the research protocol according to the parameters they wish to analyze.

Authors originally suspected personality type, namely extroversion, may alter how participants respond to research group size; however, personality measures did not reveal any statistically significant relationships with gait spatiotemporal parameters. The lack of findings may be partly due to researcher presence carrying a greater effect on gait spatiotemporal parameters than personality characteristics during simple over ground walking tasks. As well, previous studies examining personality and gait found relationships with kinematics not examined in the current study, such as trunk range of motion^12^.

The current study does have some limitations. Firstly, per each trial, participants were instructed to walk back and forth four times across a distance of 9.3 m marked out on a laboratory floor; therefore, participants experienced a total of three turns. A longer walkway will reduce the frequency of turning and may help elicit a more continuous walking pattern; however, recent research has revealed turning comprises a significant portion of daily walking^23^. Research has also suggested a total walking distance of 6.0 m with room for acceleration zones, speeding up and slowing down, or a total distance of 9.1 m is adequate for reliably determining preferred walking speed in healthy young adults^24,25^. Additionally, it was important that our in-laboratory environment closely mimic those used in other laboratory-based gait analyses. Secondly, participants’ “normal” movement during gait may have been inhibited by interference from wearing the inertial sensors on their chest, waist, wrists and feet; however, inertial measuring units are widely praised for their ability to minimize impediments typical of other motion capture systems^26^. Indeed, studies determining the difference between movement in-laboratory and real-life have sought answers using inertial measuring units^27^. Thirdly, the familiarization trial was consistently completed with our group of two-researchers present. While this may have elicited greater comfort with this condition during testing, the researchers were necessary to ensure participants could adequately execute the trial while no one was present during the no research group trial. During the familiarization trial, the researchers were also careful not to intentionally watch the participant’s walk, unlike during the small research group and the large research group trials. Additionally, participants were told that the trial was for familiarization purposes only. Fourth, while efforts were made to enhance the number and expertise of the research members, more senior researchers with more notable biomechanics expertise may elicit a stronger awareness of being judged and consequently alter movement to a greater degree. Consistent number of researchers in a large research group may also influence outcomes. Lastly, it is also important to note that the Big Five personality questionnaire, while validated, is a self-report tool and is therefore subject to bias^28^.

In conclusion, spatiotemporal parameters of gait are influenced by the number of researchers present but do not correspond to personality type. While most human movement research studies occur in a laboratory setting under strict observation, participants prove to alter their movement according to the number of researchers present. Because this alteration in spatiotemporal parameters is greater with more researchers present, it is suggested that research studies consider the number of researchers present during data collection, to acknowledge the impact of these outside sources and how they may affect the measured movement. Likewise, in teaching institutions where there is regularly a keen presence of student observation, extra effort should be made to keep the participant comfortable and their movement as regular as possible. Ideally, participants would walk the same in both an observational and a real-world setting, for inferences during in-laboratory projects to be properly interpreted to daily movement. Future research should examine whether different gait parameters require different time lengths of familiarization, as well as study the effects of researcher presence in a more heterogeneous population.

## Methods

### Participants

The present study recruited a sample of 56 college-aged men and women (37 females, 19 males, 21.4 ± 1.0 years, 169.4 ± 13.1 cm, 72.6 ± 16.9 kg). All participants provided written informed consent by reading and signing a consent form approved by the Institutional Review Board of Auburn University. To participate in this study, participants were required to be: (1) between 19 and 29 years of age, (2) without neural or musculoskeletal injury in the past 6 months, (3) regularly physically active and (4) without pain or discomfort during normal walking at the time of assessment. Participants were also assessed for asymmetric gait, through a Symmetry Index calculation, first proposed by Robinson et al.^29^. The Symmetry Index was calculated for each of the five examined variables, measuring differences between the left and right sides using Eq. (1)^29^:

1 $$SI = \frac{{\left| {X_{L} - X_{R} } \right|}}{{0.5*\left( {X_{L} + X_{R} } \right)}}*100\%$$

If the Symmetry Index was greater than or equal to 100%, participants were considered asymmetric. All participants were considered symmetric for each of the five measured spatiotemporal parameters.

### Data collection and analysis

All methods were conducted in accordance with the protocol approved by Auburn University’s Institutional Review Board. Participants were first instructed to complete two questionnaires concerning their health history and personality (Big Five Personality Test)^30^. Please see Table 2 for personality scores. Then participants’ height and mass were recorded by field measurements. Participants were instrumented with 6 wireless inertial sensors (Opal V1, APDM Inc. Sampling Frequency 128 Hz, Portland, OR, USA) secured to each wrist and foot, as well as the sternum, and lumbar (L5) region. Each participant was instructed to walk back and forth across the laboratory floor at their typical speed, for a total of 4 lengths (each length = 9.3 m) completing a 180° turn at the end of each length, before stopping at the original starting point. Upon visual inspection, the final gait cycle of each trial was affected by the participant slowing down in preparation for the end of the trial; thus, all spatiotemporal parameters means and standard deviations were calculated after removing the final stride of the collection for each trial. Turning steps are not included in the detection algorithm for gait with the use of the APDM Mobility Lab software (ML, APDM, Inc., Portland, OR, USA), so only straight-line gait was used for analysis.

**Table 2:** Means and standard deviations for all personality scores (mean ± standard deviation).

Personality scores	
Extroversion	23 ± 7
Agreeableness	33 ± 5
Conscientiousness	29 ± 7
Neuroticism	24 ± 7
Openness	25 ± 5

All Big Five scores range from 0 to 40, with higher scores indicating individuals are stronger suited with that specific trait.

Participants completed a familiarization trial first (with two researchers present, but not observing intently), and then completed three trials under different walking conditions: (1) with no researchers present, (2) with 2 researchers present, and (3) with 6–10 researchers present. To ensure participants could correctly complete the protocol, the order of condition was randomized following the familiarization practice trial. Data collection took place in the Locomotor Movement Control Laboratory of Auburn University. The group of researchers exited and entered the laboratory according to condition requirements, with valid and nonchalant excuses to distract participants from the changing of conditions. Besides comparing spatiotemporal parameters between research group conditions, parameters were also measured within trial to see how the potential effects of researcher presence changed over the course of a trial. To do so, the first and last few strides of the trial were averaged for analysis, after removal of the final stride. Specifically, in trials where participants completed 12 or fewer gait cycles (10.7%, 14.3%, and 21.4% of total sample for the no research group, small research group and large research group, respectively), the first 2 and last 2 gait cycles were used to represent the first and last portions of the trial, whereas in trials where participants completed more than 12 gait cycles, the first 3 and last 3 gait cycles were used. Data for the left and right side were averaged as the participants were previously deemed symmetric. All calculations were conducted with custom algorithms in MATLAB (R2018a, The MathWorks, Inc., Natick, MA, USA).

## Researchers

Our researchers were introduced as “experts” in the biomechanics field. Only the participant was in the lab during the no research group. The small research group included two research authors from the present study. The large research group consisted of a total of 6–10 researchers. Besides the two research authors from the small research group, additional personnel for the large research group included undergraduate, master’s, and doctoral students who worked in movement analysis laboratories. Participants were made aware of the biomechanical expertise of the researchers. Researchers were given clipboards with a sham questionnaire pertaining to the participant and their walk, to simulate an intensive research laboratory setting and to create a sense that the participant was being evaluated. For the large research group, 90.2% (n = 46) of participants had a total number of 8–10 researchers present, and 9.8% (n = 5) had a total number of 6–7 researchers present.

### Spatiotemporal parameters

APDM Mobility Lab software was used to provide a range of spatiotemporal parameters of gait. The following gait measures were selected for further analysis: gait speed (m/s), cadence (steps/min), stride length (m), step duration (s), and arm range of motion (°). These gait measures were processed using manufacture software and were then exported for further analysis. Please see Table 3 for spatiotemporal parameter definitions.

**Table 3:** Definitions table for gait parameters and Big Five personality traits.

Parameter	Units	Definition
Gait speed	m/s	The forward speed of the subject, measured as the forward distance traveled during the gait cycle divided by the gait cycle duration
Cadence	steps/min	The number of steps per minute
Stride length	m	The forward distance traveled by a foot during a gait cycle, equal to two steps
Step duration	s	The duration of a step, measured as the period from initial contact of one foot to the initial contact of the opposite foot
Arm range of motion	º	The angular range of motion of arms during arm-swing. Average of the left and right sides

Big 5 trait		Definition
Extroversion		Assesses the quantity and intensity of interpersonal interactions
Agreeableness		Assesses individuals’ concern for cooperation and social harmony
Conscientiousness		Assesses organization and goal-directed behavior
Neuroticism		Assess the degree to which individuals are prone to emotional instability
Openness		Assesses individuals’ tendency to seek out new experiences

APDM definitions are from (APDM, INC) and Big Five personality definitions are cited from Allen et al.^28^.

### Statistics

Before analysis, bivariate correlations were run to examine the relationship between personality measures including extroversion, neuroticism, openness, agreeableness and conscientiousness in relation to gait speed. Similarly, another correlation was used to assess the change in spatiotemporal parameters between conditions corresponding to personality traits. Again, no statistical significance was found; therefore, personality measures were not used further in the analyses. Statistical analysis proceeded with a one-way repeated measures multivariate analysis of variance (MANOVA) to assess the differences in five spatiotemporal parameters including: gait speed, cadence, stride length, step duration and arm range of motion, between research groups. A repeated measures MANOVA was also employed to observe differences within trial from the first few strides to the last few strides of the trial. Using G*Power software (version 3.1.9.4; Kiel, Germany)^31^ and previous research, a sample size of 47 was determined to be sufficient to detect a significant difference between conditions with an effect size of 0.80 and an alpha level of 0.05. According to Mahalanobis distance, there were no multivariate outliers^32^. Data were assessed for normality through inspection of a Shapiro–Wilk normality table, and linearity was visually inspected through use of bivariate scatterplot matrices. All data were considered normal and linear. Follow-up univariate tests were completed with a new alpha level set to 0.01 following a Bonferroni adjustment to account for the multiple analyses. All statistical analyses were performed using SPSS statistical software package (IBM SPSS Statistics Version 26, SPSS Inc., Chicago, IL, USA).

## Data Availability

The data sets generated during and/or analysed during the current study are available from the corresponding author on reasonable request.

## References

[1] Studenski S (2011). Gait speed and survival in older adults. JAMA.

[2] Vickers J, Reed A, Decker R, Conrad BP, Olegario-Nebel M, Vincent HK (2017). Effect of investigator observation on gait parameters in individuals with and without chronic low back pain. Gait Posture.

[3] Kawai H (2020). Association between daily living walking speed and walking speed in laboratory settings in healthy older adults. Int. J. Environ. Res. Public Health.

[4] Takayanagi N (2019). Relationship between daily and in-laboratory gait speed among healthy community-dwelling older adults. Sci. Rep..

[5] Fritz S, Lusardi M (2009). White paper: “Walking speed: the sixth vital sign”. J. Geriatr. Phys. Ther..

[6] Middleton A, Fritz SL, Lusardi M (2015). Walking speed: the functional vital sign. J. Aging Phys. Act..

[7] Triplett N (1898). The dynamogenic factors in pacemaking and competition. Am. J. Psychol..

[8] McCambridge J, Witton J, Elbourne DR (2014). Systematic review of the hawthorne effect: new concepts are needed to study research participation effects. J. Clin. Epidemiol..

[9] McCarney R (2007). The hawthorne effect: a randomised, controlled trial. BMC Med. Res. Methodol..

[10] Cohen JL (1980). Social facilitation. Motiv. Emot..

[11] Taylor C (1995). Philosophical Arguments.

[12] Satchell L (2017). Evidence of big five and aggressive personalities in gait biomechanics. J. Nonverbal Behav..

[13] Robles-García V (2015). Spatiotemporal gait patterns during overt and covert evaluation in patients with parkinson’s disease and healthy subjects: is there a hawthorne effect. J. Appl. Biomech..

[14] Malchow C, Fiedler G (2016). Effect of observation on lower limb prosthesis gait biomechanics: preliminary results. Prosthet. Orthot. Int..

[15] Hutchinson LA, Brown MJ, Deluzio KJ, De Asha AR (2019). Self-selected walking speed increases when individuals are aware of being recorded. Gait Posture.

[16] Purser JL (2015). Walking speed predicts health status and hospital costs for frail elderly male veterans. J. Rehabil. Res. Dev..

[17] Guralnik JM (2000). Lower extremity function and subsequent disability: consistency across studies, predictive models, and value of gait speed alone compared with the short physical performance battery. J. Gerontol. A Biol..

[18] Brinkerhoff SA, Murrah WM, Hutchison Z, Miller M, Roper JA (2019). Words matter: instructions dictate “self-selected” walking speed in young adults. Gait Posture.

[19] Tilson JK (2010). Meaningful gait speed improvement during the first 60 days poststroke: minimal clinically important difference. Phys. Ther..

[20] Lemasson A, André V, Boudard M, Lippi D, Hausberger M (2015). Audience size influences actors’ anxiety and associated postures on stage. Behav. Process..

[21] Yoshie M, Kudo K, Murakoshi T, Ohtsuki T (2009). Music performance anxiety in skilled pianists: effects of social-evaluative performance situation on subjective, autonomic, and electromyographic reactions. Exp. Brain Res..

[22] Van de Putte M, Hagemeister N, St-Onge N, Parent G, de Guise JA (2006). Habituation to treadmill walking. Bio-med. Mater. Eng..

[23] Glaister BC, Bernatz GC, Klute GK, Orendurff MS (2007). Video task analysis of turning during activities of daily living. Gait Posture.

[24] Macfarlane PA, Looney MA (2008). Walkway length determination for steady state walking in young and older adults. Res. Q. Exerc. Sport.

[25] Johnson RT, Hafer JF, Wedge RD, Boyer KA (2020). Comparison of measurement protocols to estimate preferred walking speed between sites. Gait Posture.

[26] Washabaugh EP, Kalyanaraman T, Adamczyk PG, Claflin ES, Krishnan C (2017). Validity and repeatability of inertial measurement units for measuring gait parameters. Gait Posture.

[27] Kowalsky DB, Rebula JR, Ojeda LV, Adamczyk PG, Kuo AD (2019). Human walking in the real world: interactions between terrain type, gait parameters, and energy expenditure. bioRxiv.

[28] Allen MS, Greenlees I, Jones M (2013). Personality in sport: a comprehensive review. Int. Rev. Sport Exerc. Psychol..

[29] Robinson R, Herzog W, Nigg BM (1987). Use of force platform variables to quantify the effects of chiropractic manipulation on gait symmetry. J. Manip. Physiol. Ther..

[30] Goldberg LR (1992). The development of markers for the big-five factor structure. Psychol. Assess..

[31] Faul F, Erdfelder E, Lang AG, Buchner A (2007). G*Power 3: a flexible statistical power analysis program for the social, behavioral, and biomedical sciences. Behav. Res. Methods.

[32] Mertler CA, Reinhart RV (2016). Advanced and Multivariate Statistical Methods: Practical Application and Interpretation.

